# Extracellular vesicles induce protective immunity against *Trichuris muris*

**DOI:** 10.1111/pim.12536

**Published:** 2018-05-23

**Authors:** R. K. Shears, A. J. Bancroft, G. W. Hughes, R. K. Grencis, D. J. Thornton

**Affiliations:** ^1^ Faculty of Biology, Medicine and Health Wellcome Trust Centre for Cell‐Matrix Research and Manchester Immunology Group Manchester Academic Health Sciences Centre University of Manchester Manchester UK

**Keywords:** exosomes, extracellular vesicles, *Trichuris*, vaccination, vaccine, whipworm

## Abstract

Gastrointestinal nematodes, such as *Trichuris trichiura* (human whipworm), are a major source of morbidity in humans and their livestock. There is a paucity of commercially available vaccines against these parasites, and vaccine development for *T. trichiura* has been impeded by a lack of known host protective antigens. Experimental vaccinations with *T. muris* (murine whipworm) soluble Excretory/Secretory (ES) material have demonstrated that it is possible to induce protective immunity in mice; however, the potential for extracellular vesicles (EVs) as a source of antigenic material has remained relatively unexplored. Here, we demonstrate that EVs isolated from *T. muris* ES can induce protective immunity in mice when administered as a vaccine without adjuvant and show that the protective properties of these EVs are dependent on intact vesicles. We also identified several proteins within EV preparations that are targeted by the host antibodies following vaccination and subsequent infection with *T. muris*. Many of these proteins, including VWD and vitellogenin N and DUF1943‐domain‐containing protein, vacuolar protein sorting‐associated protein 52 and TSP‐1 domain‐containing protein, were detected in both soluble ES and EV samples and have homologues in other parasites of medical and veterinary importance, and as such are possible protective antigens.

## 1 INTRODUCTION

*Trichuris trichiura* is one of the most prevalent human soil‐transmitted helminths (STHs), along with the hookworms *Necator americanus* and *Ancyclostoma duodenale,* and the roundworm, *Ascaris lumbricoides*.^1^, ^2^ These gastrointestinal parasites infect roughly one in four of the world’s population, and STH infections are often associated with anaemia, stunted growth and delayed cognitive development.^3^, ^4^ *T. trichiura* can persist in the host caecum for years, and heavy infections can lead to *Trichuris* dysentery syndrome and colitis.^5^ The currently available antihelminthic drugs have limited efficacy against the major human STH species, and crucially, drug treatment does not protect against re‐infection.^6^, ^7^, ^8^, ^9^, ^10^, ^11^, ^12^ These factors emphasize the need for prophylactic vaccines against gastrointestinal nematodes.

The first step in designing an effective vaccine is to identify pathogen‐specific components that are recognized by the host immune system, leading to parasite clearance.^13^ The soluble material released by *T. muris*, known as the ES, has formed the basis of experimental vaccines in mice,^9^, ^14^, ^15^, ^16^ while EVs remain a relatively unexplored source of host protective antigens. Recently, there has been great interest in the ability of parasite‐derived EVs, namely exosomes, to stimulate and/or modulate host immunity^17^, ^18^, ^19^ and other researchers have demonstrated that vaccination with *Heligosomoides polygyrus* EVs can protect mice against a subsequent infection.^20^ EVs have been isolated from *T. muris* and *T. suis* (porcine whipworm) secretions, as well as from other parasitic nematodes such as *Brugia malayi*.^18^, ^19^, ^21^, ^22^, ^23^

Here, we show that EVs can be isolated from *T. muris* ES by differential ultracentrifugation and show that these vesicles can protect mice from a subsequent *T. muris* infection when administered as a vaccine without adjuvant. This suggests that *Trichuris* EVs are a viable source of host protective components and that administration of recombinant *Trichuris* antigens within EVs may be an effective alternative to traditional vaccines formulated with adjuvant. These studies using *T. muris* will help inform vaccine design for *T. trichiura*.

## 2 MATERIALS AND METHODS

### 2.1 Maintenance of animals and parasites

C57BL/6 (Envigo, UK) and SCID (University of Manchester) mice were maintained in individually ventilated cages at 22 ± 1°C and 65% humidity with a 12 hour light‐dark cycle. Mice had free access to food and water, and all procedures were carried out on mice 6‐8 weeks of age or older, under the Home Office Scientific Procedures Act (1986). All experiments were carried out under project licence 70/8127 and conformed with the University of Manchester Animal Welfare and Ethical Review Body (AWERB) and ARRIVE guidelines. Animals were humanely killed by CO_2_ inhalation followed by terminal exsanguination or cervical dislocation. The Edinburgh (E) strain of *T. muris* was used for all experiments and parasite maintenance was carried out as described previously.^24^

### 2.2 Isolation of EVs

ES was collected by culturing adult parasites (day 35 to 42 post‐infection) in RPMI media supplemented with 500 U/mL penicillin and 500 μg/mL streptomycin (Sigma). Supernatants from worm cultures were collected after 4 and 18 hours and were centrifuged at 720 *g* for 15 minutes to separate the eggs (pellet) from the ES (supernatant). Supernatants were filtered using a 0.22 μm filter (Millipore) to remove cellular debris and microvesicles, and EVs were isolated by ultracentrifugation at 100 000 *g* for 2 hours in polyallomer tubes (Beckman Coulter). The EV pellet was washed by ultracentrifugation at 100 000 *g* for 2 hours in PBS. EV pellets were resuspended in 2 mL PBS and stored at −20°C until required.

### 2.3 TEM analysis of EV samples

Samples were transferred to formvar‐carbon‐coated EM grids and negatively stained with 2% (w/v) uranyl acetate. Samples were imaged using a Tecnai BioTwin microscope, at 100 Kv under low‐dose conditions. Images were recorded using a Gatan Orius CCD camera at 3.5 Å/pixel. ImageJ v1.46r (National Institute of Health) was used to view images and to add scale bars.

### 2.4 Dynamic light scattering (DLS) of EVs

Dynamic light scattering was used to measure the size distributions of the EV preparations. DLS measurements were performed using a Zetasizer Nano S (Malvern) at a controlled temperature of 25°C. Three measurements of 13 averages were taken and the number distribution of particles is reported.

### 2.5 Proteomic analysis of EVs

Preparation of EV samples for tryptic digestion was carried out as described by Marcilla and colleagues^25^ and mass spectrometry analysis was carried out as described previously.^26^ The results were analysed using Scaffold Proteome Software (Scaffold, USA) and the exclusive unique peptide count was displayed for each protein (criteria set to 95% protein threshold, 50% peptide threshold, minimum 2 peptides identified). Proteins identified in two out of the three samples were listed. The SignalP Server version 4.1 (http://www.cbs.dtu.dk/services/SignalP/, Technical University of Denmark) was used to predict whether proteins had signal peptides. The protein content of *T. muris* EVs was also compared to that of *T. muris* ES^14^ from which it was purified.

### 2.6 Vaccination studies

All vaccination studies were carried out in male C57BL/6 mice. Prior to each vaccination study, the amount of protein in each EV sample was measured using a bicinchoninic acid assay kit. Mice were vaccinated subcutaneously with 3 μg of material (either lysed or whole vesicles), followed by a second vaccination with 1.5 μg of material 14 days later (in 100 μL PBS). The sham group was vaccinated subcutaneously with 100 μL PBS only. A positive control group was included, whereby mice were vaccinated subcutaneously with 30 μg ES, followed by 15 μg of ES 14 days later (formulated in 1:1 dilution with Imject alum, Sigma). Alum vaccinations were prepared by adding adjuvant dropwise to the antigen preparation and were incubated for 40 minutes on a 360° rotator. All vaccinations were carried out using a 25G needle (BD Microlance). Mice were infected with 25 *T. muris* eggs 14 days after the second vaccination and were sacrificed at day 32 post‐infection.

### 2.7 EV lysis and protein quantification

EVs were lysed by adding 0.1% (v/v) SDS, followed by three freeze/thaw cycles, whereby vesicles were frozen in liquid nitrogen and thawed in a 37°C water bath, with vigorous vortexing between each step. Lysis was confirmed using DLS, as described above. The amount of protein in each sample was measured using a bicinchoninic acid assay, according to the manufacturer’s instructions. Protein concentration was used to standardize EV vaccinations.

### 2.8 Antiparasite IgG1 and IgG2a/c ELISAs

Antiparasite IgG1 and IgG2a/c ELISAs were carried out as described previously.^27^

### 2.9 SDS‐PAGE and western blotting

SDS‐PAGE was carried out as described previously.^28^ Electrotransfer of proteins from polyacrylamide gels to nitrocellulose membrane was carried out using an XCell IITM semiwet Blot Module run at 35 V for one hour with 20% (v/v) methanol, 1 × NuPAGE^®^ transfer buffer (Invitrogen). Membranes were blocked with 5% skimmed milk (Marvel) in Tris‐buffered saline‐Tween (TBST; 10 mmol/L Tris‐base/150 mmol/L NaCl/0.1 % (v/v) Tween‐20, pH 8.0) for 30 minutes. Membranes were probed with serum (1:300 dilution in TBST) overnight, and bound antibody was detected using an anti‐mouse IgG (whole molecule) alkaline phosphatase conjugated antibody (1:10 000 dilution in TBST, Sigma). Membranes were washed in TBST between each of these steps. Immunoblots were revealed using chromogenic substrates, BCIP (5‐bromo‐4‐chloro‐3‐indolyl‐phosphate, 100% v/v in dimethylformamide) and nitro blue tetrazolium (NBT, 70% v/v in dimethylformamide) in a 1:2 ratio in TBST.

## 3 RESULTS

### 3.1 Characterization of EVs isolated from *T. muris* ES

**Figure 1:**
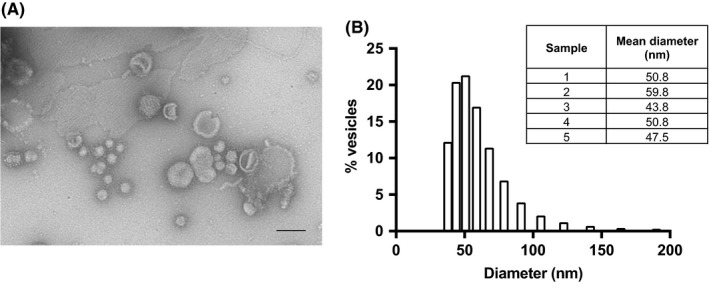
Characterization of EVs isolated from *T. muris* ES. A, TEM analysis of vesicles isolated from *T. muris* ES. Scale bars denote 100 nm, and image is representative of three preparations. B, shows the size profile of a typical *T. muris* EV sample, as measured by DLS. Table shows mean particle diameter for several samples

EVs were isolated from *T. muris* ES by ultracentrifugation at 100 000 *g* for 2 hours. Pelleted material was viewed by transmission electron microscopy, and a heterogeneous population of cup‐shaped vesicles, approximately 30‐100 nm in diameter, was observed (Figure 1A). DLS analysis also confirmed that the majority (96.5%) of vesicles isolated from *T. muris* ES were between 30 and 100 nm in diameter and this was consistent between samples (Figure 1B). The size and shape of these EVs are typical of exosomes.^29^, ^30^

Proteomic analysis revealed the presence of 125 proteins within *T. muris* EV samples (Table S1). A number of known exosome markers were identified, including tetraspanins (tetraspanin 9 and TSP‐1 domain‐containing protein), heat shock proteins, enolase, Rab proteins, and apoptosis‐linked gene 2 interacting protein X 1 (Alix, Table 1, references.^19^, ^30^ These data suggest that the vesicles isolated by ultracentrifugation of ES are likely to be exosomes. Proteomic analysis also revealed that 23% of EV proteins were not present in *T. muris* ES and 76% of these proteins lack a signal peptide (68% of total EV proteins, Table S1).

**Table 1:** List of exosome markers identified within *T. muris* EV samples

Accession number	Protein	Mw (kDa)	No. of peptides		
			Sample 1	Sample 2	Sample 3
TMUE_s0037005100	Tetraspanin 9	43	0	5	4
TMUE_s0070003500	TSP‐1 domain‐containing protein	46	3	5	3
TMUE_s0177000800	Heat shock protein 70	71	4	9	5
TMUE_s0014013200	Heat shock protein 90	81	2	6	2
TMUE_s0203001300	Small heat shock protein	16	0	2	6
TMUE_s0102000900	Enolase	48	3	5	2
TMUE_s0163002000	Ras protein Rab 11B	31	0	2	2
TMUE_s0078002300	Apoptosis‐linked gene 2 interacting protein X 1 (Alix)	122	0	2	0

The protein content of *T. muris* EVs was analysed by mass spectrometry. Table shows known exosome markers identified within *T. muris* EV samples. Mw = molecular weight. No. of peptides = number of unique peptides identified in each EV sample.

### 3.2 Vaccination with *T. muris* EVs can induce protective immunity without adjuvant and protection is dependent on intact vesicles

**Figure 2:**
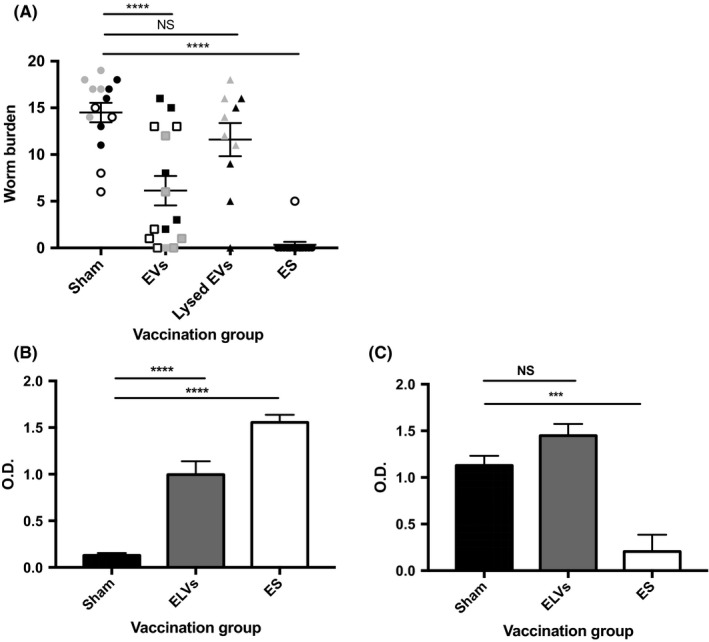
Vaccination with *T. muris* EVs induces a reduction in worm burden and a mixed Th1/Th2 response. Male C57BL/6 mice, n = 10 to 15 per group, were subcutaneously vaccinated with whole or lysed EVs, followed by a second vaccination 14 days later. The sham vaccination group received two saline injections, while the positive control group received two vaccinations with ES depleted of EVs. Mice were infected with 25 *T. muris* eggs by oral gavage. A, shows the worm burden at 32 days post‐infection. The data are pooled from three independent experiments (black, white and grey symbols indicate separate experiments). The IgG1 and IgG2a/c serum antibody responses to ES depleted of EVs were measured by ELISA and are displayed in B and C, respectively. The mean O.D. value (reading at 405 and 490 nm) for each vaccination group (sham, EV or ES vaccinated mice) is shown at 1:320 (IgG1) and 1:40 (IgG2a/c) serum dilution. Error bars show SEM, ****P < .0001, ***P < .001, NS = nonsignificant

In order to investigate whether *T. muris* EVs contain antigenic material capable of stimulating protective immunity, male C57BL/6 mice were subcutaneously vaccinated with 3 μg of isolated EVs, followed by 1.5 μg 14 days later (vaccinations were formulated without adjuvant). Mice were infected with 25 *T. muris* eggs, an infection dose that would ordinarily progress to chronicity, and worm burdens were assessed 32 days post‐infection. Vaccination with *T. muris* EVs resulted in a statistically significant reduction in worm burden compared to the sham vaccination group (vaccinated with PBS only, *P *= .0001, Figure 2A). Importantly, the mean worm burden for mice vaccinated with lysed EVs was similar to that of the sham vaccination group (*P *= .0754, Figure 2A), demonstrating that intact vesicles are required to stimulate protective immunity.

### 3.3 Vaccination with EVs boosts IgG1 serum antibody response to soluble ES components

Antiparasite IgG1 and IgG2a/c serum antibodies are often used as surrogate markers of resistance/chronicity during *T. muris* infection.^31^ The serum IgG1 and IgG2a/c antibody response against ES depleted of EVs was measured for each vaccination group. Significantly higher IgG1 antibody levels were measured for the EV vaccination group compared to the sham vaccination group (*P = *.0001, Figure 2B). High levels of antiparasite IgG2a/c, were also measured for the EV vaccination group (Figure 2C), which may suggest that EV vaccinated mice mount a mixed Th1/Th2 response, or perhaps that the infection was expelled more slowly compared to the ES vaccination group. High levels of antiparasite IgG1 (Figure 2B) and low levels of antiparasite IgG2a/c (Figure 2C) were detected for the ES vaccination group, confirming that successful vaccination stimulates Th2 immunity, while high levels of antiparasite IgG2a/c antibodies were measured for the sham vaccination group, confirming that low‐dose infection naturally primes for chronicity (Figure 2C).

### 3.4 Identification of EV components targeted by serum IgG antibodies following vaccination

**Figure 3:**
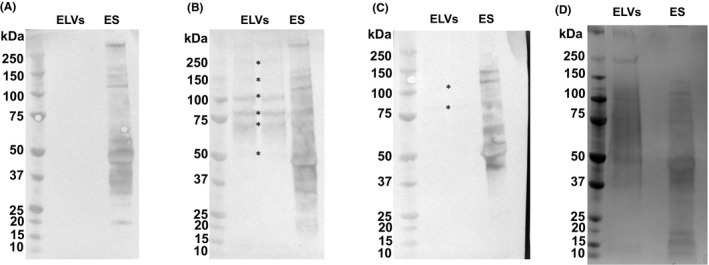
Western blots showing anti‐EV and anti‐ES serum IgG response for sham, EV and ES vaccination groups. For each blot, EV and ES components were separated by SDS‐PAGE. Samples were electrotransferred onto nitrocellulose membrane and this was probed with sera from the sham A, EV B, or ES C, vaccination groups. Bound antibody was detected using an anti‐mouse IgG (whole molecule) alkaline phosphatase antibody, and proteins were visualized using BCIP and nitro blue tetrazolium. *indicates major EV components recognized by sera. D, shows EV and ES material separated by SDS‐PAGE. Left of each panel shows molecular weight markers in kDa

Western blotting was performed to investigate which EV and ES components are recognized by serum IgG antibodies following vaccination of mice with PBS (sham), EVs or ES and subsequent *T. muris* infection (Figure 3A‐C). Infection alone does not generate IgG antibodies against EV material (Figure 3A), however, vaccination with EVs primes for IgG antibodies that target a range of EV components between 50 and 200 kDa in size (indicated by asterisks in Figure 3B). Sera collected from the ES vaccination group contained IgG antibodies that target 80 and 100 kDa EV components (indicated by asterisks in Figure 3C). Sera taken from all three groups also recognized a wide range of ES components (Figure 3A‐C).

Figure 3D shows SDS‐PAGE separation of EV and ES material. Bands corresponding to 100, 80 and 70 kDa EV components were excised from the gel, since these were the most prominent bands in Figure 3B. The protein composition of these bands was determined by mass spectrometry (Table 2). Vacuolar protein sorting‐associated protein 52, VWD and vitellogenin N and DUF1943‐domain‐containing protein and TSP‐1 domain‐containing protein were among the proteins identified within these regions (Table 2). These proteins were also identified within the soluble portion of ES, confirming that although there are differences between the protein content of *T. muris* ES and EV samples, the two are not mutually exclusive. Antigenic homologues of VWD and vitellogenin N and DUF1943‐domain‐containing protein and TSP‐1 domain‐containing protein have been identified in other parasites, and as such these are possible protective antigens.

**Table 2:** Possible identities of EV components targeted by IgG antibodies following vaccination

Accession number	Protein	Band 1	Band 2	Band 3	Mw (kDa)
aTMUE_s0245000500	VWD and vitellogenin N and DUF1943‐domain‐containing protein		9	17	190
aTMUE_s0093001800	Vacuolar protein sorting‐associated protein 52	5	4		164
aTMUE_s0070003500	TSP‐1 domain‐containing protein			3	39
aTMUE_s0189001400	Neurogenic locus notch protein		3		53
TMUE_s0015006300	Peptidase M8 domain‐containing protein		4		60
TMUE_s0037004100	Conserved hypothetical protein	4			51
aTMUE_s0022000400	Na+ K+ ATPase alpha subunit 1			3	118
TMUE_s0117002800	Trypsin and CUB domain‐containing protein	3			69
TMUE_s0093001400	Nicastrin family protein			3	78
TMUE_s0106000600	Moesin:ezrin:radixin 1	3			64
TMUE_s0011007600	Anoctamin			2	96
TMUE_s0060000200	Prominin domain‐containing protein			2	75
TMUE_s0059000500	Neurogenic locus notch protein			2	62
TMUE_s0320000100	Neurogenic locus notch protein 2			2	49

Bands corresponding to 100 (Band 1), 80 (Band 2) and 70 kDa (Band 3) were excised from the SDS‐PAGE gel shown in Figure 3D, and their protein content was analysed by mass spectrometry. The proteins identified within these bands are listed. The number of unique peptides identified for each protein is displayed (criteria set to 95% protein threshold, 50% peptide threshold).

a Indicates proteins identified within ES depleted of EVs (as reported in^14^).

## 4 DISCUSSION

The vesicles isolated from *T. muris* ES fit the size and shape characteristics for classification as exosomes, and previously described exosome markers (including tetraspanins, heat shock proteins and Alix)^30^ were identified within these samples. Mass spectrometry analysis showed that the majority of *T. muris* EV proteins lack a signal peptide (68%) and that there was significant overlap between the protein content of EVs and ES (77% of EV proteins were identified in ES). This suggests that EVs may be an important mechanism by which these proteins are released into the external environment. Similarly, Marcilla and colleagues reported significant overlap between the protein content of *F. hepatica* and *E. caproni* ES and EV samples.^25^

Here, we show that vaccination with *T. muris* EVs can induce protective immunity against a subsequent *T. muris* infection. The protection afforded by these EV vaccinations was variable; sterile immunity was achieved for some individuals, while others developed chronic infection. This has also been reported for *H. polygyrus* EV vaccinations, although in these experiments, mice were vaccinated with 10 μg of material, boosted twice with 2 μg material, and all vaccinations were formulated with alum adjuvant,^20^ whereas the vaccinations performed here contained less material and were formulated without adjuvant. Effective EV vaccination without adjuvant has been shown for other pathogen systems highlighting the inherent immunogenicity of EVs containing protein antigens compared to proteins or peptides alone.^32^, ^33^ While future work assessing the capacity of adjuvants on increasing protection levels of EV vaccination will be informative, the key observation here is that delivery of antigens in the form of EV alone induces protection. Indeed, the protective properties of *T. muris* EVs are dependent on intact vesicles, as vaccination with lysed EVs did not protect mice from a subsequent infection. Vaccine research using liposomes and microparticles may offer insight into why EVs make effective vaccines.^34^ It has been suggested that encapsulating antigens in lipid spheres protects them from degradation and enables slow release of antigen over time.^34^, ^35^, ^36^ In addition, Fifis and colleagues have demonstrated that 40‐50 nm‐sized nanoparticles are preferentially taken up by DEC205^+^ CD40^+^ CD86^+^ murine DCs compared to larger particles of up to 2 μm in size.^37^ Therefore it is reasonable to suggest that presentation of *Trichuris* proteins within EVs makes them better suited for uptake by antigen presenting cells, thus increasing their antigenicity. This should be explored further as encapsulating recombinant or purified native *Trichuris* antigens within EVs may be a viable alternative to traditional vaccinations formulated with adjuvant.

The data presented here show that vaccination with EVs boosts IgG1 antibody production against soluble ES proteins. This may be explained by the extensive overlap between proteins identified in EV samples and ES depleted of EVs (Table S1). Coakley and colleagues report similar findings, demonstrating that vaccinating mice with *H. polygyrus* exosomes prior to infection boosted antibody response to ES depleted of EVs, and that rats vaccinated with *H. polygyrus* EVs make antibodies against EV and ES material in the absence of infection.^20^ We measured high levels of antiparasite IgG2a/c in all of the EV vaccinated mice (Figure S1), and as such, found no correlation between worm burden and antiparasite IgG2a/c production. This suggests that EV vaccinated mice mount a mixed Th1/Th2 response to a low‐dose *T. muris* infection.

The sera of EV vaccinated mice recognize a number of components that are enriched within EV samples, demonstrating that these components are antigenic. The strongest antibody response was directed towards 100, 80 and 70 kDa components. Figure 3D shows SDS‐PAGE separation of the lysed EV material and mass spectrometry analysis of the protein content within these regions revealed a number of potential antigens. These include VWD and vitellogenin N and DUF1943‐domain‐containing protein, vacuolar protein sorting‐associated protein 52, and TSP‐1 domain‐containing protein, which are among the most abundant EV proteins (Table S1). Eichenberger and colleagues also identified these proteins within *T. muris* EVs.^23^ Although antibody responses may not reflect protection, the therapeutic value of related proteins has been demonstrated in other helminths,^34^, ^35^, ^36^, ^37^, ^38^, ^39^, ^40^, ^41^ suggesting that these proteins are major candidates for protective antigens.

Vitellogenin proteins isolated from the ES of gravid adult female *Litomosoides sigmodontis,* a filarial nematode of rodents, and *Ostertagia ostertagi,* an intestinal nematode of cattle, have been identified as novel vaccine candidates using immunoscreening and proteomics approaches.^38^, ^39^ Vitellogenin proteins have also been identified as potential vaccine candidates for ectoparasites, such as ticks and mites.^40^, ^41^, ^42^, ^43^ There are no published reports relating to vacuolar sorting protein‐associated protein 52; however, there appear to be homologues of this protein in other tricephalic parasites, including *Trichuris* and *Trichinella* species (Table S2). The TSP‐1 domain‐containing protein could also be a promising immunogenic candidate, given that *S. mansoni* TSP proteins have shown great potential in preclinical and Phase I clinical trials.^44^, ^45^ It is interesting to note that infection alone does not generate antibody responses against EVs or their contents, at least as assessed by Western blotting; however, this may reflect the sensitivity of the assay.

This is the first example of successful vaccination against a *Trichuris* parasite using EVs, and the first example of an EV vaccination formulated without adjuvant. Recent reports have demonstrated that vaccination with *H. polygyrus* EVs can protect mice against a subsequent infection, while vaccinating mice with *E. caproni* EVs can improve the clinical outcome of infection.^20^, ^46^ There are also a number of examples of protective immunity induced by vaccination with EVs derived from host cells, for example, vaccinating CBA/J mice with EVs collected from splenic DCs pulsed with *T. gondii* antigens before pregnancy induced protective immunity in pups, resulting in fewer brain cysts and lower mortality following congenital exposure.^47^ Similarly, del Cacho and co‐workers demonstrated that immunizing chickens with EVs derived from DCs pulsed with *Eimeria* parasites lead to reduced mortality, intestinal inflammation and faecal oocyst shedding.^48^ Martin‐Jaular and co‐workers also reported a protective role for reticulocyte‐derived EVs containing *Plasmodium yoelii* material, showing that vaccination with these EVs stimulated IgG antibodies capable of binding infected red blood cells, with 83% of mice surviving an otherwise lethal *P. yoelii* infection. Previously described *S. mansoni* vaccine candidates have also been identified in EV samples,^49^, ^50^, ^51^, ^52^, ^53^ supporting the data presented here, which suggest that helminth EVs may be an important source of protective material.

In conclusion, the data presented here show that vaccination with *T. muris* EVs can protect mice against a subsequent *T. muris* infection, and these vaccinations boost antibody response to ES depleted of EVs. A number of potential immunogenic candidates were identified by Western blotting; these include VWD and vitellogenin N and DUF1943‐domain‐containing protein, vacuolar protein sorting‐associated protein 52 and TSP‐1 domain‐containing protein. Future work should investigate recombinant forms of these proteins as protective antigens and explore opportunities for EVs to boost their antigenicity.

## CONFLICT OF INTEREST

The authors have no conflict of interests including financial interests in any company or institution.
