# Supplementary figures and images for: Extracellular vesicles induce protective immunity against Trichuris muris
Source: Parasite Immunol. 2018 May 23;40(7):e12536. doi: 10.1111/pim.12536 (PMC6055854; doi:10.1111/pim.12536)

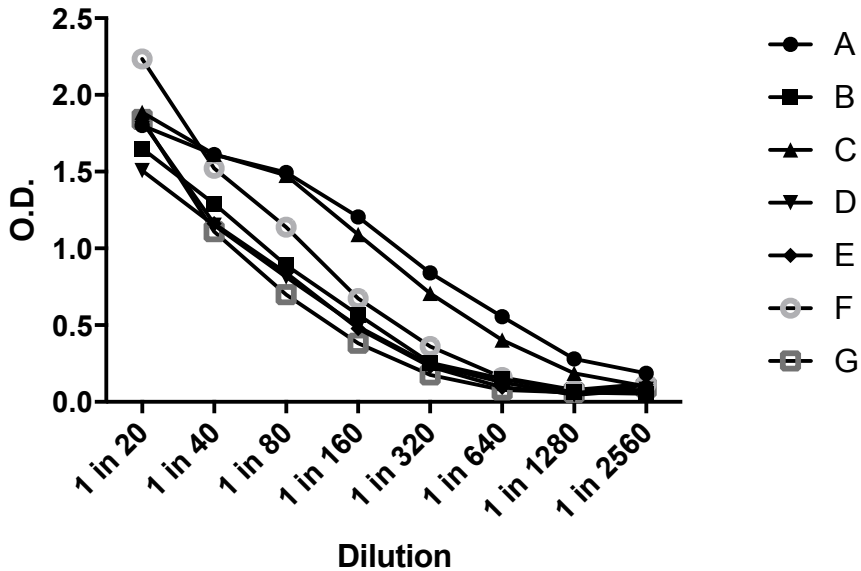

Supplement: Supplementary file 1 [file PIM-40-na-s001.pdf]
