# Supplementary material for: Extracellular vesicles induce protective immunity against Trichuris muris
Source: Parasite Immunol. 2018 May 23;40(7):e12536. doi: 10.1111/pim.12536 (PMC6055854; doi:10.1111/pim.12536)
Supplement: Supplementary file 2 [file PIM-40-na-s002.pdf]

	Accession number	Protein	Mw (kDa)	Sample 1	Sample 2	Sample 3	Identified in T. muris ES (Y/N)	Signal peptide (Y/N)
1	TMUE_s0245000500	VWD and Vitellogenin N and DUF1943-domain containing protein	202	15	52	41	Y	Y
2	TMUE_s0134000500	CAP domain containing protein	32	8	10	7	Y	Y
3	TMUE_s0049005700	Protein crumbs	382	9	17	11	Y	N
4	TMUE_s0004007100	Trypsin domain containing protein	107	7	15	8	Y	Y
5	TMUE_s0052001100	Hypothetical protein	38	6	8	7	Y	Y
6	TMUE_s0093001800	Vacuolar protein sorting associated protein 52	175	7	10	9	Y	N
7	TMUE_s0175000100	Pfam-B 9093 domain containing protein	34	8	10	8	Y	N
8	TMUE_s0003000700	Histone H4	11	2	3	6	Y	N
9	TMUE_s0029005700	Actin	42	8	7	8	Y	N
10	TMUE_s0029007100	Beta tubulin	50	6	8	11	N	N
11	TMUE_s0140001200	ABC transporter, ATP binding protein	327	4	7	5	N	N
12	TMUE_s0071005500	Angiotensin converting enzyme	153	5	7	11	Y	N
13	TMUE_s0208001100	CUB and Ldl recept a and Trypsin domain containing protein	94	7	9	7	N	N
14	TMUE_s0201000900	Pfam-B 9093 domain containing protein	39	3	7	10	Y	Y
15	TMUE_s0028000200	Phosphoenolpyruvate carboxykinase GTP	80	6	7	3	Y	N
16	TMUE_s0033001500	CAP domain containing protein	40	4	7	8	Y	Y
17	TMUE_s0175000200	Pfam-B 9093 domain containing protein	37	4	5	8	Y	Y
18	TMUE_s0091002200	Fasciclin domain containing protein	81	7	9	6	Y	Y
19	TMUE_s0177000800	Heat shock protein 70	71	4	9	5	Y	N
20	TMUE_s0005010900	Neurogenic locus notch protein	367	6	2	5	Y	Y
21	TMUE_s0026005200	Hypothetical protein	11	3	4	2	N	N
22	TMUE_s0003006100	Hypothetical protein	225	2	6	10	Y	Y
23	TMUE_s0033006300	Hypothetical protein	40	5	5	6	Y	Y
24	TMUE_s0094001000	Glyceraldehyde 3 phosphate dehydrogenase	41	7	7	5	Y	N
25	TMUE_s0004006800	Trypsin domain containing protein	129	2	7	3	Y	Y
26	TMUE_s0022002100	Clathrin heavy chain	193	0	5	8	N	Y
27	TMUE_s0046006500	Notch 2	40	3	5	5	N	N
28	TMUE_s0042005600	Motile sperm domain containing protein	24	3	4	6	Y	N
29	TMUE_s0173000900	Delta protein 4	54	4	8	5	Y	N
30	TMUE_s0002015700	Vitellogenin N and VWD and C8 and DUF1943 domain containing	354	0	6	2	Y	Y
31	TMUE_s0001012400	Phosphoprotein phosphatase 1	86	4	8	5	Y	N
32	TMUE_s0085004800	Histone H3	17	0	2	3	N	N
33	TMUE_s0003006600	Pfam B 13663 domain containing protein	164	0	6	5	Y	Y
34	TMUE_s0147001700	Hypothetical protein	15	5	4	4	Y	Y
35	TMUE_s0302000300	Fructose bisphosphate aldolase class I	44	5	8	2	Y	N
36	TMUE_s0022011000	Hypothetical protein	21	2	3	2	Y	N
37	TMUE_s0119001000	Membrane metallo endopeptidase 1 like protein	173	9	4	0	Y	N
38	TMUE_s0001012300	Hypothetical protein	39	4	6	5	Y	Y
39	TMUE_s0228001300	Na+:K+ ATPase alpha subunit protein	114	2	4	2	N	N
40	TMUE_s0062004700	Ubiquitin domain containing protein	95	2	0	3	Y	N
41	TMUE_s0052006000	Hypothetical protein	54	2	6	2	N	Y
42	TMUE_s0023009900	Peptidase family M13 containing protein	119	0	3	4	N	N
43	TMUE_s0008012300	Conserved hypothetical protein	42	3	4	2	N	Y
44	TMUE_s0049001500	Trypsin domain containing protein	139	0	4	7	Y	N
45	TMUE_s0037005100	Tetraspanin 9	43	0	5	4	Y	N
46	TMUE_s0053002900	Conserved hypothetical protein	107	2	8	0	N	N
47	TMUE_s0117003000	Eukaryotic translation elongation factor 1A	49	3	7	4	Y	N
48	TMUE_s0014013200	Heat shock protein 90	81	2	6	2	Y	N
49	TMUE_s0060006700	Protein eyes shut	81	2	6	2	N	N
50	TMUE_s0027006600	ASP domain containing protein	44	2	5	3	Y	N
51	TMUE_s0102000900	Enolase	48	3	5	2	Y	N
52	TMUE_s0106000600	Moesin:ezrin:radixin 1	69	3	8	0	Y	N
53	TMUE_s0019006300	Hypothetical protein	39	3	5	4	Y	Y
54	TMUE_s0008014600	Hypothetical protein	34	4	5	2	Y	N
55	TMUE_s0311000100	Neurogenic locus notch protein 1	52	2	3	3	N	N
56	TMUE_s0157001800	Trans 2 enoyl coenzyme A reductase	32	2	2	4	Y	N
57	TMUE_s0037004100	Conserved hypothetical protein	54	2	6	4	N	Y
58	TMUE_s0053003800	Trypsin domain containing protein	33	4	4	0	Y	Y
59	TMUE_s0022000400	Na+,K+ ATPase alpha subunit 1	118	3	6	3	Y	N
60	TMUE_s0037003800	NADP dependent malic enzyme, mitochondrial	71	0	10	2	Y	N
61	TMUE_s0327000100	Pfam-B 9093 domain containing protein	39	0	4	6	Y	Y
62	TMUE_s0042008400	Peptidase M2 domain containing protein	40	2	5	4	Y	N
63	TMUE_s0070003500	TSP-1 domain containing protein	46	3	5	3	Y	N
64	TMUE_s0024002900	Hypothetical protein	32	3	3	2	Y	Y
65	TMUE_s0203001300	Small heat shock protein	16	0	2	6	N	N
66	TMUE_s0049001600	Trypsin domain containing protein	215	0	2	4	Y	N
67	TMUE_s0120000600	Tubulin alpha chain	50	0	5	5	Y	N
68	TMUE_s0028001200	VAB 10a protein	829	3	0	3	Y	Y
69	TMUE_s0060000200	Prominin domain containing protein	76	2	3	3	Y	Y
70	TMUE_s001000830	Onchocystatin	17	4	3	3	N	N
71	TMUE_s0036001600	Conserved hypothetical protein	35	2	4	3	N	N
72	TMUE_s0117002800	Trypsin and CUB domain containing protein	71	2	4	3	Y	N
73	TMUE_s0201000800	Conserved hypothetical protein	51	0	3	2	Y	N
74	TMUE_s0191000800	Trypsin domain containing protein	79	0	5	2	Y	Y
75	TMUE_s0005001100	Kunitz protease inhibitor	25	2	4	3	Y	Y
76	TMUE_s0006000700	Solute carrier family 2, facilitated glucose	62	0	4	2	Y	N
77	TMUE_s0281000600	CBM 14 and TIL domain containing protein	202	2	6	0	Y	N
78	TMUE_s0116002200	Ubiquitin associated and SH3	41	0	6	2	Y	N
79	TMUE_s0064002700	Hypothetical protein	27	3	3	4	Y	N
80	TMUE_s0037003400	Pathogenesis protein 1B	41	3	2	4	Y	N
81	TMUE_s0189001400	Neurogenic locus notch protein	54	2	2	0	Y	Y
82	TMUE_s0012010600	EGF domain containing protein	78	2	5	2	Y	N
83	TMUE_s0014006600	Motile sperm domain containing protein	15	3	4	0	Y	Y
84	TMUE_s0086000700	78 kDa glucose regulated protein	73	2	3	0	Y	Y
85	TMUE_s0009007500	14-3-3 protein	28	5	5	0	Y	N
86	TMUE_s0015008200	Protein jagged 2	19	2	4	2	N	N
87	TMUE_s0031000300	ADP,ATP carrier protein, heart:skeletal muscle	48	3	2	2	N	N
88	TMUE_s0013012700	Motile sperm domain containing protein	14	3	3	0	Y	N
89	TMUE_s0033006400	CAP domain containing protein	35	2	4	3	Y	Y
90	TMUE_s0096005200	Ubiquitin associated and SH3 protein	39	0	4	2	Y	N
91	TMUE_s0070000700	Proprotein convertase subtilisin:kexin type 4	72	3	2	0	N	Y
92	TMUE_s0008012200	Conserved hypothetical protein	34	3	4	0	N	N
93	TMUE_s0007005500	Peroxiredoxin 2	121	3	0	2	Y	N
94	TMUE_s0014004800	Lamp domain containing protein	46	2	2	0	N	Y
95	TMUE_s0001022900	Conserved hypothetical protein	40	2	4	0	Y	N
96	TMUE_s0089001100	Conserved hypothetical protein	54	0	4	5	Y	Y
97	TMUE_s0003006000	Hypothetical protein	52	0	3	5	N	N
98	TMUE_s0033002400	Dipeptidyl peptidase 1	50	2	3	3	N	Y
99	TMUE_s0006008700	Thioredoxin	22	0	2	2	Y	Y
100	TMUE_s0022008100	BTB domain containing protein	41	0	5	2	Y	N
101	TMUE_s0054001500	E3 binding and 2 oxoacid dh and Biotin lipoyl domain containing p	53	0	2	4	N	N
102	TMUE_s0038006700	32 kDa beta galactoside binding lectin	39	0	2	2	Y	N
103	TMUE_s0058002600	Hypothetical protein	25	2	3	0	Y	N
104	TMUE_s0030008500	Conserved hypothetical protein	36	0	2	3	Y	N
105	TMUE_s0005011400	Peptidyl prolyl cis trans isomerase 7	23	2	2	0	Y	N
106	TMUE_s0023004600	Pfam-B 9093 domain containing protein	27	0	3	3	Y	N
107	TMUE_s0131003100	Prominin domain containing protein	87	0	2	2	N	N
108	TMUE_s0081001900	Protein disulfide isomerase	55	0	2	2	Y	Y
109	TMUE_s0015006100	Peptidase M8 domain containing protein	50	2	5	0	Y	N
110	TMUE_s0114001900	Hypothetical protein	21	2	3	2	N	N
111	TMUE_s0163002000	Ras protein Rab 11B	31	0	2	2	N	N
112	TMUE_s0058002100	Conserved hypothetical protein	26	2	2	0	Y	N
113	TMUE_s0122001100	Pol poly protein	43	2	2	0	Y	N
114	TMUE_s0002004000	Pfam-B 18698 and PLAT and DCX domain containing protein	118	0	2	2	Y	N
115	TMUE_s0291000100	Prominin domain containing protein	36	0	2	4	Y	N
116	TMUE_s0012010900	Neurogenic locus notch protein	43	2	3	0	Y	Y
117	TMUE_s0131003400	Hypothetical protein	23	2	2	0	Y	N
118	TMUE_s0005004200	Malate dehydrogenase	39	0	2	2	Y	N
119	TMUE_s0036001700	Conserved hypothetical protein	37	0	2	2	Y	N
120	TMUE_s0104001300	Hypothetical protein	10	3	2	0	Y	N
121	TMUE_s0119002100	Galectin	31	2	2	0	Y	N
122	TMUE_s0004012300	LIM domain containing protein	24	0	2	3	Y	N
123	TMUE_s0041006400	Hypothetical protein	33	0	2	3	Y	Y
124	TMUE_s0034006900	T complex protein 1 subunit beta	58	0	2	2	Y	N
125	TMUE_s0172001800	CH domain containing protein	41	0	2	2	Y	N
