# Supplementary material for: Extracellular vesicles induce protective immunity against Trichuris muris
Source: Parasite Immunol. 2018 May 23;40(7):e12536. doi: 10.1111/pim.12536 (PMC6055854; doi:10.1111/pim.12536)
Supplement: Supplementary file 3 [file PIM-40-na-s003.pdf]

**Protein**	**Organism**	**Query coverage (%)**	**Identity (%)**
Vacuolar sorting associated protein 52	*T. trichiura*	26	80
Vacuolar sorting associated protein 52-like protei	*T. pseudospiralis*	26	58
Vacuolar sorting associated protein 52-like protei	*T. patagoniensis*	26	59
Vacuolar sorting associated protein 52-like protei	*T. papuae*	26	59
Vacuolar sorting associated protein 52-like protei	*T. zimbabwensis*	26	59
Vacuolar sorting associated protein 52-like protei	*T. nelsoni*	26	58
